# Acoustic Observation of Living Organisms Reveals the Upper Limit of the Oxygen Minimum Zone

**DOI:** 10.1371/journal.pone.0010330

**Published:** 2010-04-30

**Authors:** Arnaud Bertrand, Michael Ballón, Alexis Chaigneau

**Affiliations:** 1 Institut de Recherche pour le Développement, UMR212 EME, CRH, Sète, France; 2 Instituto del Mar del Perú, Esquina Gamarra y Gral. Valle s/n, Lima, Peru; 3 Institut de Recherche pour le Développement, IPSL/LOCEAN, UPMC/CNRS/IRD/MNHN, Paris, France; National Oceanic and Atmospheric Administration/National Marine Fisheries Service/Southwest Fisheries Science Center (NOAA/NMFS/SWFSC), United States of America

## Abstract

**Background:**

Oxygen minimum zones (OMZs) are expanding in the World Ocean as a result of climate change and direct anthropogenic influence. OMZ expansion greatly affects biogeochemical processes and marine life, especially by constraining the vertical habitat of most marine organisms. Currently, monitoring the variability of the upper limit of the OMZs relies on time intensive sampling protocols, causing poor spatial resolution.

**Methodology/Principal Findings:**

Using routine underwater acoustic observations of the vertical distribution of marine organisms, we propose a new method that allows determination of the upper limit of the OMZ with a high precision. Applied in the eastern South-Pacific, this original sampling technique provides high-resolution information on the depth of the upper OMZ allowing documentation of mesoscale and submesoscale features (e.g., eddies and filaments) that structure the upper ocean and the marine ecosystems. We also use this information to estimate the habitable volume for the world's most exploited fish, the Peruvian anchovy (*Engraulis ringens*).

**Conclusions/Significance:**

This opportunistic method could be implemented on any vessel geared with multi-frequency echosounders to perform comprehensive high-resolution monitoring of the upper limit of the OMZ. Our approach is a novel way of studying the impact of physical processes on marine life and extracting valid information about the pelagic habitat and its spatial structure, a crucial aspect of Ecosystem-based Fisheries Management in the current context of climate change.

## Introduction

Oceans include vast areas called oxygen minimum zones (OMZs) where subsurface layers are depleted in dissolved oxygen (DO) [1]. OMZs are separated from the well-oxygenated surface mixed-layer by strong vertical DO gradients forming the oxycline. These OMZs contribute to 25–75% of oceanic N_2_O production [2], a potent greenhouse gas, which influences the Earth's heat budget and depletes stratospheric ozone [3]. OMZs are generally the site of intense denitrification [4] and have notable effects on the distribution and mortality of marine organisms [5], [6], [7]. Although a few species of zooplankton, mesopelagic fish, and squids have adapted their metabolism to temporarily (through diel vertical migration) or permanently inhabit OMZs, most marine species limit their distribution to the surface oxygenated layer [5], [6]. In response to global warming and direct anthropogenic influences, OMZs of the World Ocean are expanding [5], [8]–[10]. The upper limit of OMZs is rising and consequently, the vertical extent of the well-oxygenated surface layer shrinks, constraining the vertical habitat of epipelagic organisms. Intensification of oxygen-poor and acidic conditions could severely impact marine communities e.g. by (i) shrinking the available habitat, (ii) diminishing the capacity of plankton to develop calcium carbonate skeletons, (iii) eliminating species from metazoans to fish predators or (iv) hampering the spawning success of fish resources [5], [7], [11]–[16]. Upwelling regions are particularly vulnerable given that they encompass the largest OMZs [1] and sustain ∼20% of worldwide fish captures [17].

The oxycline, which delimits the top of the OMZ, forms a sharp barrier for living organisms intolerant to hypoxia. It is also the site of the most intense particulate matter remineralization, a process contributing to maintain the underlying OMZ [18]. Monitoring the spatial extent of the harshly acidic OMZ is crucial for assessing the effects of climate change on physical, chemical and biological mechanisms of marine ecosystems [8], [15], [19], [20]. However, since DO direct measurements require the deployment of oxygen sensors at discrete stations, the amount of available DO observations is relatively low [8], [19]. High-resolution observation of the spatiotemporal variability of the oxycline cannot be achieved on a regional scale with conventional methods (including Argo profilers or underwater autonomous vehicles). Here we propose a new method for estimating the lower oxycline depth at very high spatiotemporal resolution using the vertical distribution of epipelagic organisms (mainly zooplankton and small pelagic fish) estimated using acoustics. Acoustic instruments are widely used to detect fish, zooplankton and other objects far beyond the optical range [21]. We exploited this capability to determine the lower vertical extension of the epipelagic community (VEEC), constrained by the OMZ, using bi-frequency acoustic data collected continuously along survey tracks.

The method is applied in the highly productive region of the eastern South Pacific off Peru, which supports the world's largest monospecific fisheries, the Peruvian anchovy (*Engraulis ringens*) [22], and encompasses one of the most intense and shallow OMZ [1], [22]–[24]. In this region, intense upwelling cells lead to a coastal oxycline depth of usually less than 25 m, which strongly impacts marine life [20]. We use this information to estimate, in particular, the habitable volume for the Peruvian anchovy.

## Results

**Figure 1:**
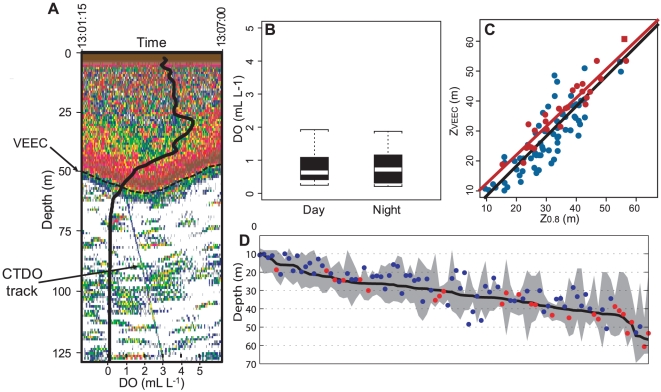
Acoustic detection of the VEEC during the ‘*Filamentos 2008*’ survey. **A**. Example of acoustic echogram showing a CTDO track and the VEEC. The superimposed black solid line is the corresponding DO vertical profile (mL L^−1^, lower axis). **B**. Box plot of DO concentration at *Z_VEEC_* according to the diel period. **C.** Relationship between *Z_VEEC_* and *Z_0.8_* for the 25 stations with detectable CTDO tracks on echograms (full red circles; the full red square in the upper right corner corresponds to the station presented in **A**) and the other 71 CTDO stations (full blue circles); Red and black solid lines correspond to the linear regression for the 25 stations with detectable CTDO tracks and for all the 96 stations, respectively. **D**. Vertical range of the lower oxycline (shaded area) for all 96 stations ranked toward increasing *Z_0.8_* (black solid line); full dots represent *Z_VEEC_* for the 25 stations with “visible” CTDO tracks (red) and the other 71 CTDO stations (blue).

**Figure 2:**
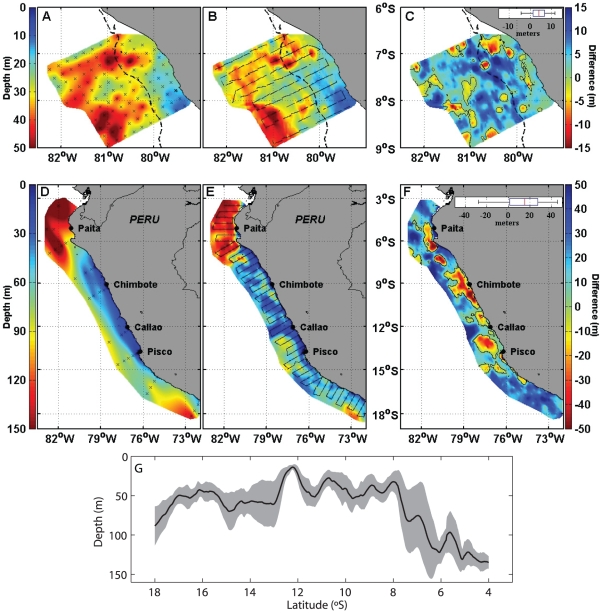
Spatial distribution of the upper OMZ depth. Upper OMZ depth estimated from *Z_0.8_* determined from CTDO measurements (**A**) and Niskin bottles profiles (**D**) and *Z_VEEC_* estimated from acoustic measurements (**B**, **E**). Black crosses indicate the position of hydrographic stations (**A**, **D**) whereas black lines indicate acoustic tracks (**B, E**). **C** and **F** differences between *Z_0.8_* and *Z_VEEC_*; black contours correspond to a null difference; boxplots of the differences are displayed on the upper right part of (**C**) and (**F**). Upper panel (**A, B, C**) corresponds to the ‘*Filamentos 2008’* survey; dotted lines indicate the depth of the 200 m bottom depth. Lower panel (**D, E, F**) corresponds to the ‘*Pelagic 2005*’ survey. Left colour-bars correspond to figures (**A, B, D, E**) while right colour-bars correspond to figures (**C, F**). **G**. Meridional variation of *Z_VEEC_* averaged between the coast and 200 km offshore during the ‘*Pelagic 2005*’ survey (black solid line) and corresponding ± one standard deviation (grey shaded area).

The lower limit of the VEEC (*Z_VEEC_*), was defined as the depth at which 98% of accumulated acoustic echoes occurs (Fig. 1A, see Materials and Methods). We first worked on acoustic data from the ‘*Filamentos 2008’* survey realized off Peru in February 2008 (Fig. 2B, Table 1). During this survey, 113 hydrographic stations were sampled (Fig. 2A) to acquire vertical profiles of physical-biogeochemical parameters using a conductivity-temperature-depth probe equipped with a dissolved oxygen sensor (CTDO). Among these 113 stations, 96 included parallel acoustic measurements for which 25 showed visible CTDO tracks within the acoustic echograms such as the one displayed in Figure 1A. Those 25 reference casts allowed highly precise measurement of the DO concentration at *Z_VEEC_*.

**Table 1:** Survey characteristics.

Survey	‘Filamentos 2008’	‘Pelagic 2005’
Vessel	R/V Olaya from IMARPE	R/V Olaya from IMARPE
Start and End dates	Feb. 05, 2008–Feb. 20, 2008	Feb. 20, 2005–Apr. 04, 2005
Covered area	06°30′S–08°02′S	3°29′S–18° 03′S
Sampled hours	264	600
Echosounder	Simrad EK 60	Simrad EK500
Frequencies	38 and 120 kHz	38 and 120 kHz
DO measurement	CTD Sea Bird Electronic 911	Niskin bottle
N° DO profiles	113	33
N° of DO profiles visible on the echogram	25	0
N° of DO profiles with ≥300 acoustic data available within a 5 km range	71	20

Based on these reference DO profiles and the concomitant echograms, we determined that the mean DO concentration at *Z_VEEC_* was 0.80 mL L^−1^, regardless of the diel period (Fig. 1B; ANOVA day-night effect: F[_1_,_23_] = 0.0005, p = 0.98) or the distance from the coast (ANOVA offshore-inshore effect: F[_1,23_rsqb; = 0.3518, p = 0.56). The linear relationship between *Z_VEEC_* and the depth of the DO isovalue of 0.80 mL L^−1^ (*Z_0.8_*) was highly significant (Table 2; Fig. 1C). We extended the analysis to the remaining 71 CTDO profiles for which CTDO casts were not visible on the concomitant echograms and obtained similar results (Table 2; Fig. 1C).

**Table 2:** Linear regressions summary.

Survey	Case	*y*	*x*	Slope	Intercept	*n*	*F*	*p*	*R^2^*
Filamentos 2008	CTDO visible on echogram	Z_VEEC_	Z_0.8_	0.95	2.96	25	203.5	<0.0000	0.90
Filamentos 2008	CTDO not visible on echogram	Z_VEEC_	Z_0.8_	0.95	−1.19	71	177.1	<0.0000	0.72
Filamentos 2008	All CTDO with echogram	Z_VEEC_	Z_0.8_	1.00	−1.68	96	324.4	<0.0000	0.78
Filamentos 2008	CTDO visible on echogram	Z_VEEC_	Z_bot.oxy._	0.87	2.59	25	108.5	<0.0000	0.83
Filamentos 2008	CTDO not visible on echogram	Z_VEEC_	Z_bot.oxy._	0.88	−1.94	70*	154.6	<0.0000	0.70
Filamentos 2008	All CTDO with echogram	Z_VEEC_	Z_bot.oxy._	0.93	−2.39	95*	272.5	<0.0000	0.75
Pelagic 2005	Use of Niskin bottles	Z_VEEC_	Z_0.8_	0.90	7.0	20	101.1	<0.0000	0.85

*note that it was not possible to estimate Z_bot.oxy_ for one coastal station since the corresponding CTDO profile did not reach the base of the oxycline.

We defined the lower oxycline as the vertical region comprised between the depth of the maximum DO vertical gradient and the deepest level where vertical DO gradient value was weaker than −0.2 mL L^−1^ m^−1^ (bottom oxycline) (see Material and Methods). The *Z_0.8_* and most *Z_VEEC_* were included in the lower oxycline region (Fig. 1D) and *Z_VEEC_* was highly significantly correlated with the bottom oxycline (Table 2). To test for the robustness of *Z_VEEC_* as a proxy of the upper OMZ boundary we repeated the analysis with echograms and *in situ* DO profiles collected during the routine acoustic survey *‘Pelagic 2005’* performed in February-April 2005 along the entire Peruvian coast (Fig. 2E, Table 1). During this survey, 20 DO profiles were acquired from Niskin bottles (Fig. 2D), while acoustic data were also recorded. The linear correlation between *Z_VEEC_* and *Z_0.8_* estimated from Niskin bottle casts was again highly significant (Table 2, Fig. S1).

These results show that *Z_VEEC_* estimated from acoustic data is a robust proxy of the lower oxycline depth - upper limit of the OMZ. Spatially interpolated maps of the upper OMZ as estimated from *Z_VEEC_* are very consistent with those obtained from DO profiles (Fig. 2) with a mean difference lower than 5 m for the ‘*Filamentos 2008*’ survey (Fig. 2C). Since the use of Niskin bottles (7 discrete levels on the first 150 m of the water column, see Materials and Methods) only provides a rough estimation of *Z_0.8_*, this mean difference is increased for the *‘Pelagic 2005’* survey.

**Figure 3:**
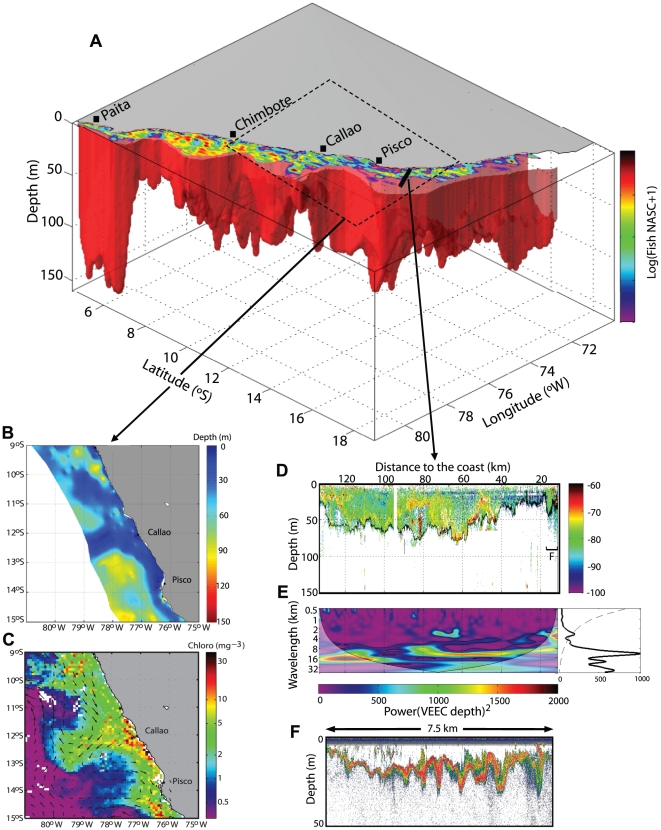
Volume of anchovy habitat along the Peruvian coast. **A**. Volume (red volume) estimated by integrating *Z_VEEC_* over the area occupied by the cold coastal water and its mixing with adjacent water masses [24] during ‘*Pelagic 2005’* survey. The upper part of the volume shows anchovy distribution estimated during the same survey. **B**. Zoom of the study area between 9°S and 15°S (black dotted rectangle) showing a region of shallower *Z_VEEC_*. This region corresponds to a mesoscale filament associated with strong westward geostrophic currents and high chlorophyll concentration as observed from geostrophic currents (black quivers) from satellite altimetry AVISO product and chlorophyll-a concentration (colours, in mg m^−3^) from satellite SeaWiFS data for the same time period (**C**). The black solid line south of Pisco in (**A**) corresponds to the transect presented in (**D**) showing the echogram and the *Z_VEEC_* (black solid line) along this transect. **E**. Wavelet power spectrum (in m^2^) of *Z_VEEC_* in this transect showing the presence of mesoscale (≥10 km) and submesoscale (**F**) Features.

Our method provides high resolution spatial maps of the vertical limit of the oxygenated habitat (Figs. 2B, 2E) which allows estimation of the available volume for organism habitat. We applied this to the Peruvian anchovy whose horizontal distribution is limited by the offshore extension of the upwelled cold coastal water and its mixing with adjacent water masses [25], [26]. Anchovy volume of habitat can thus be estimated by integrating *Z_VEEC_* over the horizontal area occupied by these water masses, and determined according to [26]. Using this approach we estimated the available volume of anchovy habitat to be 9187 km^3^ along the Peruvian coast during the ‘*Pelagic 2005*’ survey (Fig. 3A). Thus, by applying this methodology to any acoustic survey, we are now able to describe the patterns of variability of this volume of habitat at different spatial scales.

During the *‘Pelagic 2005’* survey, the upper limit of the OMZ exhibited large-scale meridional fluctuations varying from a local maximum of ∼70–80 m south of 17°S, to an average value of ∼50 m between 16°S and 8°S, and progressively deepened northward to reach 140 m at 4°S (Figs. 2E,G, 3A). These patterns agree well with previous estimates based on historical CTD casts [27]. Cross-shore large-scale variations are also observed, with minimum Z*_VEEC_* values in coastal regions where upwelling takes place and deeper OMZ in the offshore ocean (Figs. 2B, 2E), in agreement with [27].

Superimposed on these large-scale patterns, mesoscale features of few tens of kilometres can be easily identified from Z*_VEEC_* measurements. For instance, during *‘Pelagic 2005*’ survey eddy-like mesoscale structures were centred at 18°S–73°W and 14°S–77°W (Figs. 2E, 3A). Mesoscale structures observed from *Z_VEEC_* fit well with those observed from satellite data. For instance, during the *‘Pelagic 2005’* survey, the region of shallower *Z_VEEC_* values (∼15 m depth) perpendicular to the coast at 12°S–13°S (Fig. 2E,G) corresponds to a filament associated with strong westward geostrophic currents and high chlorophyll-a concentration (Fig. 3B,C).

Furthermore, the upper limit of the OMZ shows typical high-frequency variations of a few kilometres such as around 7°S (Figs. 2B,2E). These submesoscale features are clearly observed along cross-shore acoustic transects (Fig. 3D,F) and this high-frequency and small-scale variability was confirmed using wavelet analyses. As an illustration, the wavelet analysis of *Z_VEEC_* along a cross-shore transect realized south of Pisco during *‘Pelagic 2005’* survey showed significant scales of 3 km very close to the coast and between ∼60–70 km from the coast and a dominant ∼10 km scale is observed all along this particular transect (Fig. 3E).

Thus, compared with conventional methods, the spatial resolution of the upper limit of the OMZ is drastically increased using acoustic data. For example, *Z_VEEC_* acquired at a frequency of one ping per second corresponds to a ∼5 m resolution along the vessel track for a cruising speed of 10 knots. Ground truth measurements from DO sensors however will always be needed to validate acoustic estimation and access to the whole DO vertical structure.

## Discussion

Based on acoustic observations, our method allows for a precise determination of the upper limit of the OMZ with high spatiotemporal resolution (Figs. 1, 2). The estimated boundary (0.8 mL L^−1^) did not change with either the diel period (day vs. night) or the position from the coast (inshore vs. offshore). Since offshore zooplankton and fish communities strongly differ from inshore species [25], [28], our methodology does not seem to be affected by changes in the communities composing acoustic scatterers. Although migratory (adapted to hypoxia) and non-migratory communities are different, the presence or absence of migratory mesopelagic communities does not affect the defined *Z_VEEC_*. This suggests that when both migratory and non-migratory species are distributed in the shallow near-surface layer (night situation), they share the same vertical range.

The information provided by this study can be used for various scientific applications. For instance, according to the habitat-based hypothesis [20], [25], variations in the range of habitat constrain the extension-contraction of fish distribution and determine their abundance if favourable or unfavourable conditions last long enough to influence their population dynamics. A decrease in the vertical range of habitat can exclude species from a region and/or dramatically modify predator-prey relationships [6], [13], [29], [30]. Organisms intolerant to anoxia could forms dense aggregations above the oxycline, while resistant species would have access to an extended refuge area. Monitoring the volume and characteristics of the 3D habitat of pelagic resources is thus crucial to better understand their population dynamics. We applied our method to the world's most exploited fish species, the Peruvian anchovy, and estimated its available habitat for a specific survey (Fig. 3A). One might notice that the biomass of anchovy is not necessarily correlated with *Z_VEEC_*, possibly because a deepened OMZ does not necessarily mean improved foraging efficiency. Rather, a shallow OMZ would concentrate plankton for foraging fish [31]. Applying this method to historical and future acoustic surveys will allow reconstruction of time series of habitat volume and the study of the impact of habitat volume dynamics on anchovy patterns of abundance and distribution at multiple scales.

This method also allows performing integrated studies since acoustic data also provides information on most ecosystem components (see Fig. 3A for anchovy distribution) within and outside this volume, to which we can add ancillary information (satellite data, vessel monitoring system, top predator tagging, etc.). Such integrated approaches are crucial to implement the ecosystem approach to fisheries [32]. Our methodology can also be applied to other ecosystems, e.g. oceanic dead zones [5], and opens new perspectives for comprehensive multiscale studies on the impact of physical forcing on organisms.

Physical forcing at meso- and submeso- scales is increasingly suspected to play a fundamental role in the structuring and functioning of marine ecosystems [20], [33]. However, instrumental sampling and present computational resolution limit the degree to which the impact of physical dynamics on living marine resources can be studied at these scales. The proposed method based on acoustic data allows for the resolution of a large range of meso and submesoscale structures such as eddies, fronts, filaments and internal waves (Fig. 3B,D,F). Scales of patterns described in high resolution local models of eastern boundary currents [34], [35] can now be documented from acoustic measurements.

In conclusion, the proposed method (i) allows for high-resolution spatial monitoring of the upper limit of the OMZ, a parameter especially relevant for physical, biogeochemical and biological processes and interactions in the context of climate change; (ii) can be easily implemented on any vessel equipped with acoustic echosounders [36], [37]; and (iii) allows revisiting historical acoustic data for the reconstruction of spatiotemporal dynamics of the upper limit of the OMZ. This method should be applied not only in areas already known to encompass an OMZ (e.g. Eastern tropical North Pacific, Arabian Sea) but also, before fish kills were noted, in systems where hypoxia/anoxia has been apparently increasing and affecting organisms (e.g. Oregon, [9]).

## Materials and Methods

### Data collection

Field data were collected on board the 41 m long *R/V ‘Olaya’* from the Instituto del Mar del Perú (IMARPE) during two scientific surveys: a multidisciplinary specific survey ‘*Filamentos 2008*’ performed in February 2008 and a routine acoustic survey ‘*Pelagic 2005*’ performed in February-April 2005 (Table 1).

### Acoustic data

Acoustic data were collected using hull-mounted Simrad split-beams bi-frequency (38 and 120 kHz) scientific echo-sounders EK500 and EK60 (Kongsberg Simrad AS) during the ‘*Filamentos 2008*’ and ‘*Pelagic 2005*’ surveys, respectively. Survey tracks consisted of parallel cross-shore transects with a target vessel speed of 10 knots. Echosounder calibration was performed according to [38]. The water column was sampled down to depths of 250 m and 500 m for the 120 kHz and 38 kHz channels respectively. Due to the presence of noise in echograms at 120 kHz, only the first 150 m were considered in the case of the ‘*Pelagic 2005*’ survey. Only day and night periods were considered; data from twilights were removed prior to analyses since it is not possible to determine *Z_VEEC_* when mesopelagic organisms migrate through the upper limit of the OMZ. Biological sampling of organisms observed by acoustic was performed using nets. Fish and other large organisms were collected by pelagic trawl ‘Engel 124/1800’ (12 mm codend mesh). Zooplankton samples were taken with Hensen nets of 0.33 m^2^ mouth area with a 300 µm mesh, in vertical hauls between 0 and 50 m. Anchovy distribution was obtained from IMARPE routine acoustic biomass evaluation [39]–[41]. Nautical-area-backscattering coefficients (NASC or s_A_; see [42] for acoustic units) were recorded along survey tracks at each one nautical mile long georeferenced elementary distance sampling unit [21]. During the ‘*Pelagic 2005*’ survey, anchovy biomass was estimated to 16.4 million tonnes [41]. The interpolated map of anchovy NASC distribution (Fig. 3A) was obtained by ordinary kriging fitted with an omnidirectional variogram based on robust estimator from [43].

### Oceanographic data

During the *Filamentos 2008* survey, 113 Conductivity-Temperature-Depth (CTD) casts were realized along 11 cross-shore oceanographic sections using a Seabird (SBE) CTD profiler composed of an underwater unit with conductivity, temperature and pressure sensors and a SBE911*plus* V2 deck unit. This CTD-SBE911*plus* model was also equipped with a SBE43 oxygen sensor calibrated one month before the cruise by the manufacturer, who ensures a precision of 0.03 mL L^−1^. The CTD was horizontally mounted on a SBE32 carousel water sampler including 12 1.7-liter Niskin bottles. Only downward CTD casts were retained for the analysis. During the ‘*Pelagic 2005*’ survey, water samples were collected using only Niskin bottles at 0, 10, 25, 50, 75, 100, and 150 m and DO concentrations were determined using the modified Winkler method [44] with a precision higher than 0.1 mL L^−1^. Oxygen profiles from both the ‘*Filamentos 2008*’ and ‘*Pelagic 2005*’ surveys were linearly interpolated to determine the depth of the 0.8 mL L^−1^ DO level (*Z_0.8_*). Oceanographic profile data were classified according to the diel period (day-night) and their spatial distribution: inshore-offshore.

### Acoustic data processing

The epipelagic community includes mainly zooplankton, gelatinous organisms and fish. Where OMZ is present, the vertical extension of most of this community is restricted to the oxygenated surface waters (see [13] for thresholds of hypoxia for marine organisms and [45] for organisms' aggregation at the boundaries of OMZs). Below, the upper part of the OMZ is generally almost free of organisms. At dusk the mesopelagic community migrates towards the surface and mixes with the epipelagic community. At dawn they migrate vertically and take refuge in the OMZ. We defined the VEEC depth (*Z_VEEC_*) to be the depth where 98% of the cumulated sum of acoustic echoes (S_v_, Volume backscattering strength, in dB re 1 m^−1^) from the epipelagic community was reached (the cumulated sum is integrated downward from the surface). To determine the 98% threshold we used two different methods. First we tested different thresholds by 1% lag between 95 and 99% and visually scrutinized the patterns of *Z_VEEC_* for each threshold in different conditions (day-night, offshore-inshore). Lower thresholds underestimated the main limit of organisms vertical distribution, while with a too high threshold (99%) the pattern of *Z_VEEC_* was erratic in some cases (i.e. when few strong scatterers were distributed below the main boundary); 98% appeared to be the best compromise in any condition. Second, we plotted the vertical gradients of cumulated S_v_ (ΔS_v_) and observed a higher gradient (or a pick, i.e. an accumulation of organisms) in the lower part of the distribution of epipelagic community when about 98% of the cumulated S_v_ was reached (Fig. S2). *Z_VEEC_* was corrected to take into account the transducer position on the R/V hull (3.4 m below the sea surface).

Anchovy (*Engraulis ringens*), the dominant epipelagic fish in the system, have much higher target strength (∼−50 dB) than euphausiids (∼−85 dB), but fish generally occupy only a small part of the epipelagic habitat while zooplankton fills most of the space (zooplankton includes mainly crustaceans and gelatinous organisms). Therefore, to better reflect the actual distribution of the overall epipelagic community, we considered all echoes but minimized the weight of fish echoes by a factor 10^−3^ when estimating *Z_VEEC_*.

To discriminate between fish and other scatterers we applied a bi-frequency analysis. We synchronised the ping number and position between echograms using the matching ping number algorithm from Echoview (SonarData Pty. Ltd., Hobart, Tasmania, Australia). Noise due to acoustic absorption was removed by subtracting (using the linear minus algorithm from Echoview) the noise field created using a data generator algorithm based on a noise function with the form:

20*log(R)+2* α *R+offset

where *R* is the range (in m), *α* is the frequency absorption coefficient (in dB.m^−1^) and the *offset* value (in dB) is the assumed initial noise at the first metre. The *α* and *offset* values were determined by using a program written in Matlab by Paul Fernandes (Marine lab., Aberdeen, UK). Then, we used the “resample by number of pings” algorithm from Echoview to resample the bi-frequency echograms to common elementary cells of 1 ping long and 0.75 m height.

Fish with swimbladder, such as anchovy, have slightly higher backscatter at 38 than 120 kHz while zooplankton have higher backscatter at 120 kHz [46]. To ensure good separation between fish and other sources of scatter, we increased the contrast between the different scatter groups by summing the backscatter response at both frequencies [47]. Then, based on our observations, we chose a threshold value of −135 dB and used a Boolean mask (true for values above threshold) to extract fish data from other scatter and created fish and no fish echograms at each frequency. We refined fish data from fish echograms by applying a second Boolean mask to keep only the targets for which: S_v_ 120–S_v_ 38 < +2 dB. The value +2 dB has been chosen as a margin error to include situations in which a fish aggregation is more insonified by the 120 kHz beam than by the 38 kHz beam, resulting in a higher backscatter at 120 kHz than at 38 kHz.

It is important to mention that the estimation of *Z_VEEC_* was robust (<1 m except in some cases when very dense fish aggregation were present) to changes in methodology (for instance, the change in the weight of 10^−3^ which we applied to fish echoes). Generally, with the exception of situations where a high number of dense fish schools are present, *Z_VEEC_* can be estimated directly from one frequency (120 kHz and probably 200 kHz) with no imperative need for multifrequency analysis.

### Comparing *Z_VEEC_* with DO profiles

To determine the DO concentration at *Z_VEEC_* for each profile two distinct methods were used. Firstly, when the CTDO track was visible on the concomitant echogram (e.g. Fig. 1A) we precisely determined (in both the horizontal and vertical planes) the DO concentration at the depth where the CTDO cast crossed the epipelagic boundary (*Z_VEEC_*). Secondly, when the CTDO track was not visible on the concomitant echogram or when the considered profile was acquired from discrete Niskin bottles (‘*Pelagic 2005*’ survey) instead of CTDO (‘*Filamentos 2008*’ survey), such precision was not possible. In these cases, since *Z_VEEC_* is affected by sub-mesoscale oceanographic dynamics such as internal waves and can exhibit vertical displacements of tenths of meters within typical horizontal range of ∼100 m (Figs. 1A, 3F; [19]), the small scale and high-frequency variability was filtered by averaging *Z_VEEC_* over the closest 300 acoustic pings recorded within a maximum radius of 5 km from the oceanographic stations.

### Oxycline definition

The oxycline separates the well-oxygenated mixed-layer from the underlying OMZ. It can be divided into an upper and a lower oxycline. The upper oxycline extends from the base of the mixed-layer where oxygen values start to decline to the depth where DO vertical gradients reach their minimum value (Fig. S3). In contrast, the lower oxycline extends from this latter level down to the bottom oxycline or the top of the OMZ defined either by the depth where DO concentrations drop below 0.5 mL L^−1^ [48] or by the maximum depth where vertical DO gradients are weaker than −0.9 µMol kg^−1^ m^−1^ equivalent to −0.02 mL L^−1^ m^−1^ [18], [23]. In this study, the base of the lower oxycline was defined as the maximum depth between these two above criteria. In the particular case shown in 3, the base of the lower oxycline corresponds to the definition given by [18], [23].

### Wavelet analysis

The cross-shore variation and periodicity of *Z_VEEC_* during the survey ‘Pelagic 2005’ were investigated using continuous wavelet analysis which is well suited to the study of multicycle, nonstationnary phenomena, occurring over finite spatial and temporal domains [49]. The continuous wavelet transform (CWT) of the space series *d* with respect to the wavelet ψ, chosen here as the Morlet wavelet, is defined as
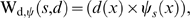
where *x* is space and ψ_s_ is the wavelet at the scale *s*. The CWT decomposes the space series into a space-wavelength space, enabling the identification of both the dominant modes of variability and how those modes vary with space. The energy distribution within the data array is computed using an adjusted wavelet power spectrum [50] defined as 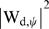, i.e., the squared transform coefficient and divided by the scale it associates. Following [51], a cone of influence (COI) is defined to remove the data *d(x)* whose wavelet transform is affected by edge effects.

## Supporting Information

Figure S1 Correlation between Z*_VEEC_* averaged over the 300 pings closest to the Niskin bottle profile and Z*_0.8_* during the routine *‘Pelagic 2005’* acoustic survey (solid line). The dotted line indicates the 1/1 slope.
(0.90 MB EPS)

Figure S2 Determination of the threshold to determine Z*_VEEC_*. Examples of vertical profiles of the cumulated Sv (red solid lines) and its vertical gradient ΔSv (blue solid lines). The corresponding dissolved oxygen (DO) profile is also shown (black solid lines). Dotted black lines indicate the intersection with 98% of cumulated S_v_.
(2.06 MB EPS)

Figure S3 Oxycline definition. Example of vertical DO profile (thick black line, upper axis) and the corresponding DO vertical gradients (thick blue line, lower axis) acquired during the *‘Filamentos 2008’* cruise. Grey shaded area corresponds to the oxycline separated into upper and lower oxycline as described in the text. The thin black line, corresponds to the depth of the maximum DO vertical gradient, and separates the upper and lower oxycline. The purple square corresponds to Z*_VEEC_* whereas the red square indicates the depth of the 0.8 mL L^−1^ level (Z*_0.8_*).
(1.24 MB EPS)
